# GPRC6A mediates Alum-induced Nlrp3 inflammasome activation but limits Th2 type antibody responses

**DOI:** 10.1038/srep16719

**Published:** 2015-11-25

**Authors:** Dagmar Quandt, Kathrin Rothe, Christoph Baerwald, Manuela Rossol

**Affiliations:** 1 Division of Rheumatology, Department of Internal Medicine, University of Leipzig, Liebigstr.20, 04103 Leipzig, Germany

## Abstract

Alum adjuvanticity is still an unknown mechanism despite the frequent use as vaccine adjuvant in humans. Here we show that Alum-induced inflammasome activation *in vitro* and *in vivo* is mediated by the G protein-coupled receptor GPRC6A. The Alum-induced humoral response *in vivo* was independent of the inflammasome because Nlrp3−/− and ASC−/− mice responded normally to Alum and blockade of IL-1 had no effect on antibody production. In contrast, Alum adjuvanticity was increased in GPRC6A−/− mice resulting in increased antibody responses and increased Th2 cytokine concentrations compared to wildtype mice. *In vitro* activation of GPRC6A−/− splenic B cells also induced increased IgG1 concentrations compared to wildtype B cells. For the first time, we show GPRC6A expression in B cells, contributing to the direct effects of Alum on those cells. B cell produced immunostimulatory IL-10 is elevated in GPRC6A−/− B cells *in vitro* and *in vivo*. Our results demonstrate a dual role of GPRC6A in Alum adjuvanticity. GPCR6A activation by Alum leads to the initiation of innate inflammatory responses whereas it is an important signal for the limitation of adaptive immune responses induced by Alum, partially explained by B cell IL-10.

Aluminum salts (Alum) are commonly used adjuvants for routine human vaccination, although its mode of action remains unclear. They induce a type 2 immune response which is characterized by the appearance of antigen-specific IgG1 and IgE antibodies in the serum (reviewed in^1^). Multiple mechanisms to explain Alums mode of action have been proposed (reviewed in^2^). One hypothesis was that Alum serves as a depot in the body from which the antigen is released slowly^3^. This has been challenged by several studies. Important findings are that the adsorbed antigen is released very fast from Alum when exposed to interstitial fluid^4^ and that stable adsorption of antigen to Alum is not necessary for the adjuvanticity of Alum^5^. More recently, a major role of the Nlrp3 inflammasome in Alum adjuvanticity was postulated^6^,^7^. The Nlrp3 inflammasome is a multiprotein complex formed by Nlrp3, apoptosis-associated speck-like protein (ASC) and caspase-1 and facilitates the release of the cytokines IL-1β and IL-18^8^. However, there is a controversy on the participation of the Nlrp3 inflammsome to Alum adjuvanticity. Some groups reported a partial contribution of the Nlrp3 inflammasome^9^ or no contribution^10^,^11^,^12^,^13^. Other hypotheses on Alum adjuvanticity include the release of danger-associated molecular patterns (DAMPs). Alum is thought to induce cell death and subsequent release of uric acid^9^ and DNA^13^ to enhance antibody responses.

Recently, our group described increased extracellular calcium acting as DAMP via the G protein-coupled receptors GPRC6A and calcium-sensing receptor (CaSR) resulting in Nlrp3 inflammsome activation in monocytes^14^. Lee *et al.* simultaneously reported the activation of the Nlrp3 inflammasome via Ca^2+^ and CaSR^15^. In addition to Ca^2+^, both receptors also sense aluminum ions^16^,^17^ and induce IL-1β in a caspase-1-, ASC- and Nlrp3-dependent manner in monocytic cells^14^. We therefore hypothesized that Alum controls antibody responses via CaSR and GPRC6A.

Here we show that Alum adjuvanticity is increased in GPRC6A−/− mice resulting in increased antibody responses and increased Th2 cytokine concentrations compared to wildtype mice. In contrast, the *in vitro* and early *in vivo* inflammatory cytokine responses triggered by Alum are decreased in GPRC6A−/− mice. We observed no participation of Nlrp3, ASC and IL-1 in Alum-induced antibody responses. We also show that splenic B cells express GPRC6A and the *in vitro* induced IgG1 is increased in B cell cultures from GPRC6A−/− compared to wildtype mice.

## Results

### Alum-induced myeloid cytokine response is decreased in GPRC6A−/− mice

**Figure 1:**
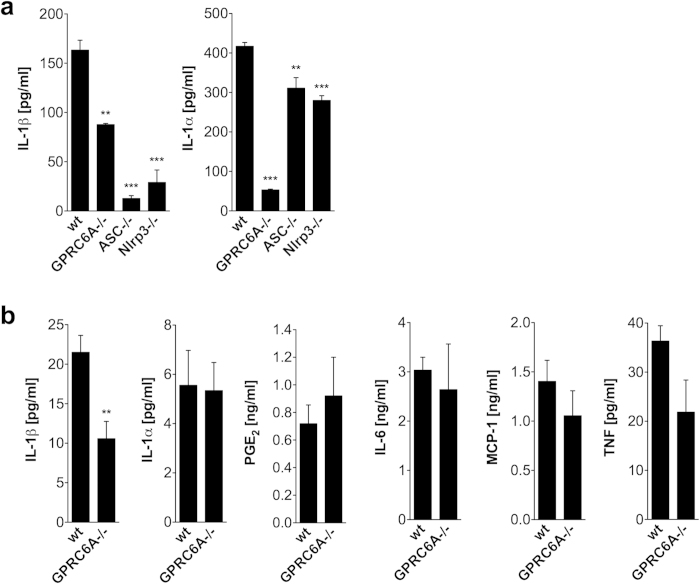
Alum-induced cytokine response is decreased in GPRC6A−/− mice. (**a**) Peritoneal macrophages from 6 wildtype (wt), 3 GPRC6A−/−, 3 ASC−/− and 3 Nlrp3−/− mice were cultured for 16 h in the presence of LPS and Alum. Cytokine concentrations were determined in the supernatant by ELISA. Statistical analysis was performed using t-test. Bars represent mean  ±  SEM. (**P < 0.01, ***P < 0.001). (**b**) Wildtype (wt) and GPRC6A−/− mice were intraperitonally injected with Ova/Alum and cytokine concentrations of IL-1β (n = 6), IL-1α (n = 9), PGE_2_ (n = 5), IL-6 (n = 5), MCP-1 (n = 5), TNF (n = 5) were determined by ELISA (IL-1β, IL-1α, PGE_2_) or CBA (IL-6, MCP-1, TNF) after 4 h. Statistical analysis was performed using t-test. Bars represent mean  ±  SEM. (*P < 0.05, **P < 0.01).

In order to assess the participation of GPRC6A in alum-induced macrophage activation, peritoneal macrophages from wild type and mice deficient of GPRC6A were isolated and *in vitro* stimulated with LPS and alum. We have shown previously, that the response to LPS or LPS/ATP is normal in macrophages of GPRC6A−/− mice^14^. Alum induced an increased IL-1β and IL-1α secretion compared to LPS alone (data not shown). As shown in Fig. 1a, Alum-induced IL-1β and IL-1α secretion is reduced in GPRC6A−/− macrophages compared to macrophages from wild type mice. Similar results were obtained for CD11b+ cells from blood and bone marrow (data not shown). In addition, Alum-induced cytokine responses in macrophages from ASC−/−, Caspase1−/− and Nlrp3−/− mice were also determined. Secretion of IL-1β is strongly reduced in ASC−/− (Fig. 1a), Nlrp3−/− (Fig. 1a) and Caspase1−/− (Supplementary Fig. S1) macrophages. Alum-induced IL-1α secretion is only minimal reduced in ASC−/− and Nlrp3−/− macrophages (Fig. 1a). These results were also obtained using ASC-, Caspase1- and Nlrp3-deficient human THP-1 cell lines (data not shown).

To assess the *in vivo* participation of GPRC6A in an early cytokine response, Ova/Alum was injected into the peritoneal cavity of wild type and GPRC6A−/− mice and cytokine concentrations in the peritoneal cavity were determined 4 and 24 hours later. As shown in Fig. 1b for 4 hours, the Alum-induced IL-1β response is diminished in GPRC6A−/− mice compared to wild type mice, whereas IL-1α, PGE_2_, IL-6, MCP-1 and TNF responses are not influenced by the loss of GPRC6A. At 24 hours, IL-1 and PGE_2_ were not detectable anymore and dramatically reduced IL-6 and similar MCP-1 and TNF responses were not different in GPRC6A ko mice (data not shown). Next we analyzed the cellular composition of the peritoneal lavage 24 hours after injection of Ova/Alum into the peritoneal cavity. No differences were observed between wildtype and GPRC6A−/− mice, neither in total cell count prior or post immunization nor in cell frequency distribution. (Supplementary Fig. S2).

### Alum adjuvanticity is increased in GPRC6A−/− mice

**Figure 2:**
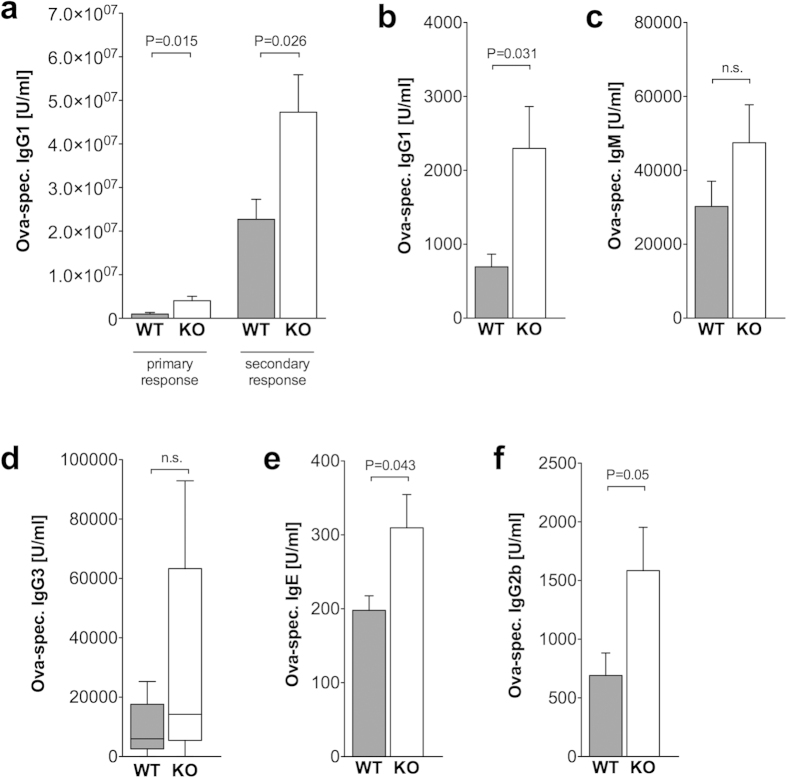
Alum adjuvanticity is increased in GPRC6A−/− mice. (**a**–**f**) 9 Wildtype (WT, gray) and 10 GPRC6A−/− (KO, white) mice were immunized with Ova/Alum by i.p. injection on day 0 and boosted on day 10. Serum samples and bone marrow samples were obtained on day 25, bone marrow supernatant was harvested on day 5 of culture, and antibody titers were measured by ELISA. (**a**–**c**,**e**,**f**) Statistical analysis was performed using t-test. Bars represent mean  ±  SEM. (**d**) Statistical analysis was performed using Mann-Whitney rank sum test. Boxes and whiskers represent 25–75 and 5–95 percentiles. For significant differences, levels of significance are given.

In order to analyze the participation of GPRC6A in the adjuvant effect of Alum *in vivo*, wildtype and GPRC6A−/− mice were immunized with Ova/Alum and sera were obtained to measure Ova-specific antibody concentrations before (day 10) and after booster immunization (day 21–25). As shown in Fig. 2a, Ova-specific IgG1 antibodies were found in increased concentrations in GPRC6A−/− mice, even in the primary antibody response at day 10 post immunization. Ova-specific IgG1 antibodies were not detectable in mice immunized with Ova alone (data not shown). Of note, the total IgG concentration in the serum of unimmunized mice was equal in wildtype and GPRC6A−/− mice (Supplementary Fig. S3). In addition, we found increased Ova-specific IgG1 antibodies in the supernatant of bone marrow cultures (day 5) from GPRC6A−/− mice compared to wildtype mice, both immunized with Ova/Alum 25 days before (Fig. 2b). We extended our analyses to Ova-specific IgM and other switched antibody isotypes in immunized mice. The concentrations of Ova-specific IgM and IgG3 antibodies were slightly increased different between wildtype and GPRC6A−/− mice, but the difference did not reach statistically significance (Fig. 2c,d). Ova-specific IgE and IgG2b antibodies were found in increased concentrations in immunized GPRC6A−/− mice compared to wildtype mice (Fig. 2e,f).

**Figure 3:**
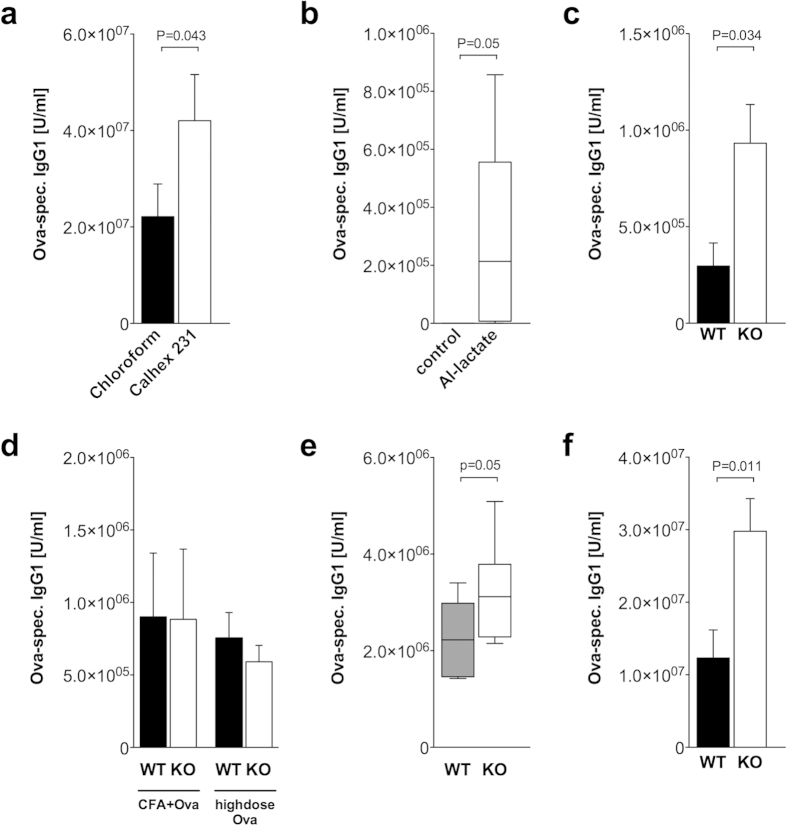
GPRC6A and CaSR are involved in Alum adjuvanticity and the effect is independet of immunization route. (**a**) Wildtype mice were either treated with Calhex231 (9 mice) or the solvent control chloroform (10 mice) and immunized with Ova/Alum by i.p. injection on day 0 and boosted on day 10. Statistical analysis was performed using t-test. Bars represent mean ± SEM. (**b**) Wildtype mice were either treated with Ova alone (6 mice) or Ova/Al-lactate (6 mice) by i.p. injection on day 0 and boosted on day 10. Statistical analysis was performed using Mann-Whitney rank sum test. Boxes and whiskers represent 25–75 and 5–95 percentiles. (**c**) 4 Wildtype (WT, black) and 3 GPRC6A−/− (KO, white) mice were immunized with Ova/Al-lactate by i.p. injection on day 0 and boosted on day 10. Statistical analysis was performed using t-test. Bars represent mean ± SEM. (**d**) Wildtype (WT, black) and GPRC6A−/− (KO, white) mice were immunized with either Ova/CFA or by s.c. injection (8 WT, 6 KO) or high dose OVA i.p. injection (10 WT, 10 KO) on day 0 and boosted on day 10. Bars represent mean ± SEM. (**e**) 8 Wildtype (WT, black) and GPRC6A−/− (KO, white) mice were immunized with Ova/Alum by i.m. injection on day 0 and boosted on day 10. Statistical analysis was performed using t-test. Bars represent mean ± SEM. (**f**) 5 Wildtype (WT, black) and GPRC6A−/− (KO, white) mice were immunized with Ova and Aluminum hydroxide by i.p. injection on day 0 and boosted on day 10. Statistical analysis was performed using t-test. Bars represent mean ± SEM. (**a**–**f**) Serum samples were obtained on day 21 and antibody titers were measured by ELISA.

CaSR-deficient mice were not available to us, therefore we tested the involvement of CaSR in Alum adjuvanticity using the specific inhibitor Calhex 231^18^. We previously showed the inhibition of Al^3+^-induced IL-1β in monocytes by Calhex 231^14^. Application of Calhex 231 in addition to Ova/Alum in wildtype mice resulted in an increased antibody response compared to the chloroform solvent control (Fig. 3a). Additionally, the LPS/Alum-induced myeloid cytokine response in peritoneal macrophages from wildtype mice is decreased when Calhex 231 is administered *in vitro* (data not shown).

Aluminum hydroxide is thought to unfold its adjuvant properties by adsorption of protein onto charged Aluminum particles in Aluminum hydroxide gels. To test whether a combination of protein and free Aluminum ions also works as an adjuvant, mice were immunized with Ova and Aluminum lactate, which is a water-soluble salt and does not form a gel. As shown in Fig. 3b, Ova in combination with Aluminum lactate also elicits an increased IgG1 antibody response when compared with Ova alone, albeit resulting in lower concentrations when compared to Aluminum hydroxide as adjuvant. In GPRC6A−/− mice, the Ova-specific IgG1 concentrations are also increased compared to wildtype mice (Fig. 3c).

Next we tested if the observed increased antibody response in GPRC6A−/− mice is specific for Aluminum-based adjuvants or is also observed with other immunization protocols. As shown in Fig. 3d, immunization of mice with Ova and the alternative adjuvant CFA led to an equal IgG1 antibody response in wildtype and GPRC6A−/− mice. Immunization with high dose Ova protein without an adjuvant also led to a comparable IgG1 antibody response in wildtype and GPRC6A−/− mice (Fig. 3d).

When Aluminum hydroxide is used in vaccines for humans, it is usually administered intramuscularly. Therefore we tested this vaccination route also in GPRC6A−/− mice. As shown in Fig. 3e, i.m. administration of Ova/Alum also resulted in increased concentrations of Ova-specific IgG1 antibodies in GPRC6A−/− mice.

All experiments were carried with Alum Imject®, which is a combination of Aluminum hydroxide and Magnesium hydroxide. To test whether the increased concentrations of Ova-specific IgG1 antibodies in GPRC6A−/− mice are not only due to the presence of Magnesium hydroxide, we used Alum consisting of Aluminum hydroxide alone. As shown in Fig. 3f, the administration of Aluminum hydroxide and Ova also resulted in increased concentrations of Ova-specific IgG1 antibodies in GPRC6A−/− mice.

### The Alum-induced IL-1 response is not required for its adjuvanticity

**Figure 4:**
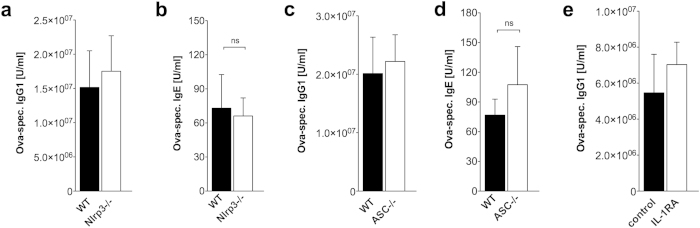
The Alum-induced inflammasome and IL-1 response is not required for its adjuvanticity. (**a**) 6 Wildtype (WT, black) and 6 Nlrp3−/− (white) mice were immunized with Ova/Alum by i.p. injection on day 0 and boosted on day 10. (**b**) 5 Wildtype (WT, black) and 9 Nlrp3−/− (white) mice were immunized with Ova/Alum by i.p. injection on day 0 and boosted on day 10. (**c**) 14 Wildtype (WT, black) and 13 ASC−/− (white) mice were immunized with Ova/Alum by i.p. injection on day 0 and boosted on day 10. (**d**,**c**) 5 Wildtype (WT, black) and 4 ASC−/− (white) mice were immunized with Ova/Alum by i.p. injection on day 0 and boosted on day 10. (**e**) Wildtype mice were immunized with Ova/Alum by i.p. injection on day 0 and boosted on day 10 and either treated with IL-1RA (n = 7) or control (n = 8). (**a**–**e**) Serum samples were obtained on day 21 and antibody titers were measured by ELISA. Statistical analysis was performed using t-test. Bars represent mean ± SEM. For significant differences, levels of significance are given.

Two cytokines, IL-1β and IL-1α, were found to be decreased in response to Alum in GPRC6A−/− mice. IL-1β is processed by the inflammasome and it was previously reported that Alum adjuvanticity depends on the inflammasome^6^,^7^. However, several studies since then reported different findings^10^,^11^,^12^,^13^. As shown in Fig. 4, we also observed no participation of Nlrp3 (Fig. 4a) or ASC (Fig. 4c) in Alum-induced increase of serum IgG1 antibody production, no participation of Nlrp3 (Fig. 4b) or ASC (Fig. 4d) in Alum-induced increase of serum IgE antibody production, and no participation of ASC in Alum-induced increase of IgG1 antibody production in bone marrow cultures (data not shown). These findings exclude a major role of inflammasome-induced IL-1β for antibody production and therefore cannot explain our finding of increased antibody concentrations in GPRC6A−/− mice. There also was no difference in spleen Tfh frequencies in immunized ASC−/− mice compared to wildtype mice (data not shown).

To ascertain the role for IL-1α, which is only minimal reduced in Alum-activated *in vitro* macrophage cultures of Nlrp3−/− or ASC−/− mice, we blocked the IL-1 receptor, which recognizes both IL-1α and IL-1β, with an IL-1R antagonist. As shown in Fig. 4e, blockade of the IL-1 receptor had no influence on Alum-induced IgG1 antibody production. These findings exclude a major role of IL-1, produced in the early response, in the observed increased IgG1 antibody production in GPRC6A−/− mice.

### Early and late cellular splenocyte contribution to enhanced antibody response in GPRC6A −/− mice

**Figure 5:**
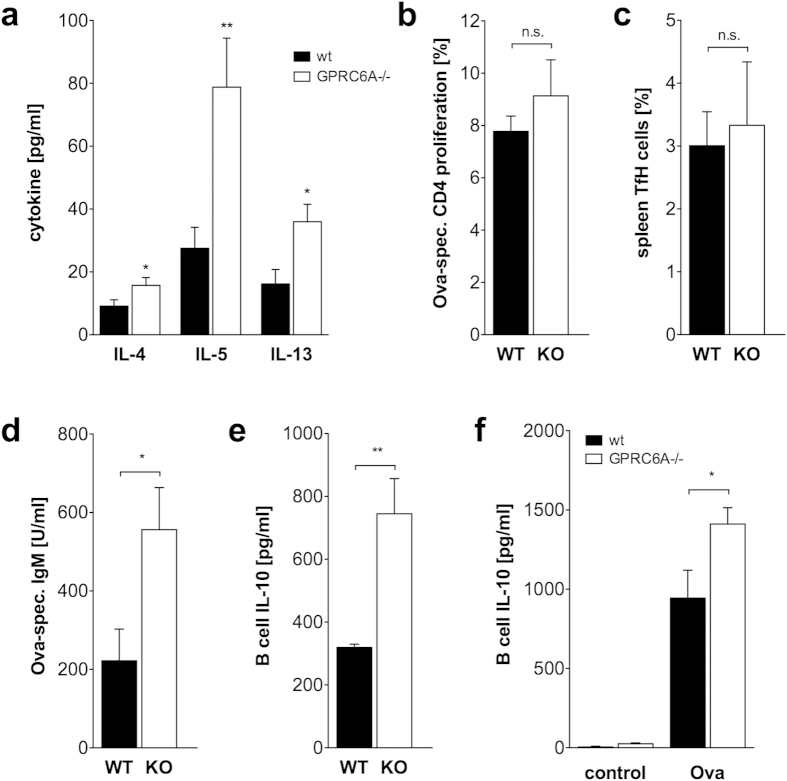
Increased Th2 cytokine production in splenocytes of GPRC6A−/− mice. (**a**) Splenocytes from 8 wildtype (wt, black) and 7 GPRC6A−/− mice (white) immunized with Ova/Alum (25 days) were *in vitro* restimulated with Ova and supernatants were harvested 3 days later. Cytokine concentrations were measured by CBA. (**b**) Splenocytes from 9 wildtype (wt, black) and 9 GPRC6A−/− mice (white) immunized with Ova/Alum (25 days) were *in vitro* restimulated with Ova and CD4+ T cell proliferation using CFSE staining was measured. (**c**) *Ex vivo* Tfh frequencies in splenocytes from 6 wildtype (wt, black) and 7 GPRC6A−/− mice (white) immunized with Ova/Alum (25 days). (**d**–**f**) Splenic B cells from 7 wildtype (wt, black) and 7 GPRC6A−/− mice (white) immunized with Ova/Alum (6 or 8 days) were *in vitro* restimulated for one day with PMA/Ionomycin (**e**) or 5 days with Ova and anti-CD40 antibodies (**d**,**f**). Antibody titers and IL-10 concentrations were measured by ELISA. (a-f) Statistical analysis was performed using t-test. Bars represent mean ± SEM. (*P < 0.05, **P < 0.01).

To analyze underlying mechanisms leading to increased IgG1 and IgE antibody production in GPRC6A−/− mice, splenocyte Th2 cell cytokine concentrations in response to Ova re-stimulation were determined. As shown in Fig. 5a, Ova-induced production of IL-4, IL-5 and IL-13 were increased in splenocyte cultures from Alum/Ova-immunized GPRC6A−/− mice compared to wildtype mice. IL-10 was not different between wildtype and GPRC6A−/− splenocytes and IL-21 was not detectable (data not shown). Proliferation of Ova-specific splenocytic CD4+ T cells from the same cultures as cytokine analyses was not different between wildtype and GPRC6A−/− mice (Fig. 5b). Spleen Tfh frequencies of immunized GPRC6A−/− mice at day 25 were comparable to wildtype mice (Fig. 5c) and total splenocyte number also not different between immunized GPRC6A−/− mice and wildtype mice (data not shown). To determine if the early IgM production by spleen B cells is already increased in GPRC6A−/− mice, animals were immunized with Alum/Ova and on day 6 and 8, B cells were sorted from splenocytes and *in vitro* re-stimulated with Ova and anti-CD40. *In vitro* production of Ova-specific IgM in response to restimulation with Ova was increased in those cultures from day 8 GPRC6A−/− mice compared to wildtype mice (Fig. 5d) but not in cultures from day 6 (data not shown). Ova-specific IgG1 was neither detectable in splenocyte cultures from day 6 nor day 8.

As shown in Fig. 5e,f, B cell-secreted IL-10 is significantly increased in B cell cultures from GPRC6A−/− mice. B cells already released elevated amounts of IL-10 after short term restimulation with PMA/Ionomycin *in vitro* demonstrating that the IL-10 production was initiated *in vivo* (Fig. 5e). Restimulation of isolated B cells with Ova and aCD40 antibodies for 5 days, additionally demonstrated the antigen-related capability of GPRC6A−/− B cells to secrete increased levels of IL-10 (Fig. 5f).

### *In vitro* activation of B cells by LPS in the presence of Alum induces increased antibody production in GPRC6A−/− B cells

**Figure 6:**
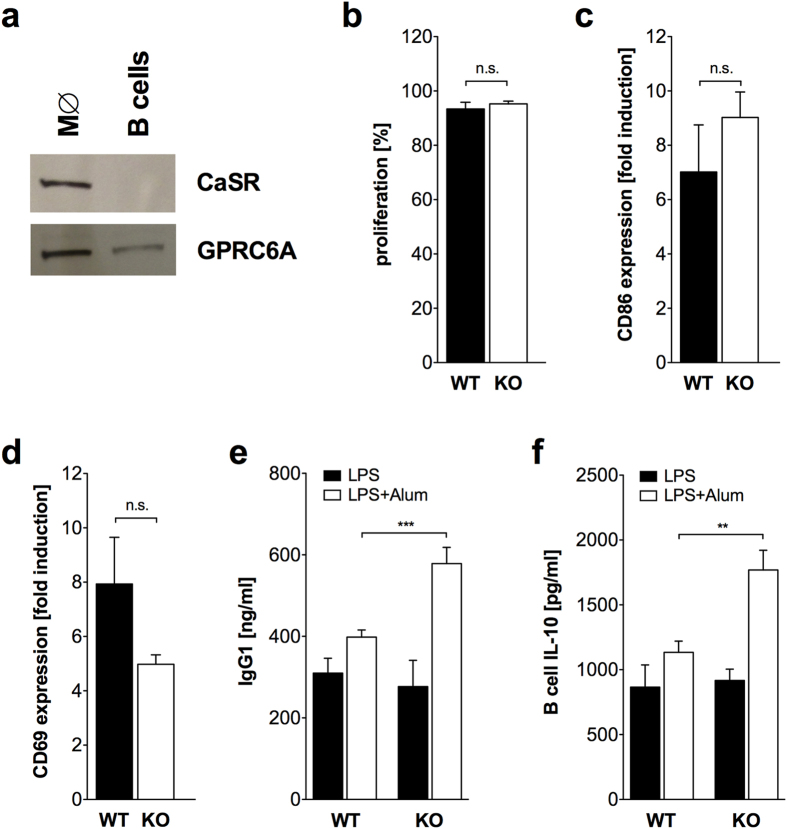
*In vitro* activation of B cells by LPS/Alum induces increased antibody production in GPRC6A−/− B cells. (**a**) Immunoblot analysis demonstrating the expression of CaSR and GPRC6A in peritoneal macrophages (M∅) and splenic B cells isolated from C57/BL6 mice. Shown is one representative experiment out of three. (**b**–**d**) Splenic B cells from 4 wildtype (WT, black) and 3 GPRC6A−/− mice (KO, white) were *in vitro* activated with LPS/Alum and proliferation on day 7 (**b**), CD86 expression on day 7 (**c**) and CD69 expression on day 1 (**d**) was analyzed by flow cytometry. Statistical analysis was performed using t-test. Bars represent mean ± SEM. (**e**) Splenic B cells from 3 wildtype (WT, black, n = 9) and 3 GPRC6A−/− mice (KO, white, n = 7) were *in vitro* activated with LPS or LPS/Alum and IgG1 concentration in the supernatant was analyzed by ELISA on day 7. Statistical analysis was performed using t-test. Bars represent mean ± SEM. (**f**) Splenic B cells from 2 wildtype (WT, black, n = 6) and 2 GPRC6A−/− mice (KO, white, n = 6) were *in vitro* activated with LPS or LPS/Alum and IL-10 concentration in the supernatant was analyzed by ELISA on day 7. Statistical analysis was performed using t-test. Bars represent mean ± SEM. (**P < 0.01, ***P < 0.001).

It was previously reported that human B cells do not express CaSR but respond to calcium via an unknown receptor^19^. As shown in Fig. 6a, mouse splenic B cells also do not express CaSR, but express GPRC6A.

Alum is able to directly activate B cells *in vitro*^20^. In order to analyze the direct effect of Alum on B cells, splenic B cells were *in vitro* polyclonally activated by LPS in the presence of Alum. Proliferation (Fig. 6b) and induction of the activation marker CD86 (Fig. 6c) and CD69 (Fig. 6d) was not different between B cells from GPRC6A−/− and wildtype mice. However, Alum+LPS induced IgG1 production was markedly increased in B cell cultures from GPRC6A−/− mice compared to wildtype mice, while B cell cultures with LPS alone did not reveal differences in IgG1 production (Fig. 6e). Alum alone did not induce IgG1 production (data not shown). IgM and IgG2b from the same cultures revealed no differences between wildtype and GPRC6A−/− mice (data not shown). Interestingly, IL-10 production was significantly increased in B cell cultures from GPRC6A−/− mice (Fig. 6f).

## Discussion

Aluminum salts are widely used as adjuvant but the mechanisms underlying its adjuvanticity remain poorly understood. Our study shows a role of the G protein-coupled receptor GPRC6A in Alum adjuvanticity but also demonstrates the dispensability of the Nlrp3 inflammasome for initiation of Th2 type immunity in Alum adjuvanticity.

Our study demonstrates the role of GPRC6A in early innate responses to Alum. IL-1β and IL-1α were produced in decreased concentrations by peritoneal macrophages deficient in GPRC6A following *in vitro* stimulation with Alum. This is not due to a general defect of GPRC6A−/− macrophages because the response to LPS and LPS/ATP is normal in these cells^14^. The *in vitro* Alum-induced IL-1β also depends on the presence of ASC, Nlrp3 and Caspase-1, as reported previously^6^,^7^,^21^,^22^. *In vivo* injection of Alum into the peritoneal cavity demonstrated that GPRC6A is necessary for roughly 50% of the produced IL-1β. The cell infiltrate in the peritoneal cavity was not influenced by GPRC6A, therefore the reduced cytokine response most likely corresponds to a decreased cytokine production and not merely to a diminished occurrence of the respective cytokine-producing cell type. These results are in line with our previously published results on the GPRC6A-dependent activation of the Nlrp3 inflammasome by Aluminum ions *in vitro* and *in vivo*^14^.

Our results suggest that Alum activates the Nlrp3 inflammasome in monocytic cells via involvement of GPRC6A. GPRC6A is able to detect free Aluminum ions^17^, however, Aluminum hydroxide is water-unsoluble, forms a gel and in this condition presumably, Aluminum ions are inaccessible to the receptor. So far, the binding of such compounds to GPRC6A has not been investigated. Flach *et al.* described that Alum crystals directly engage the plasma membrane and these contacts result in “peeling off” some crystal layers^23^. Another possibility is the phagocytosis of Alum particles and the subsequent localization of the compound in the lysosomal pathway. The acidic environment in the lysosom might result in the appearance of Aluminum ions and due to lysosomal rupture and cell death, the ions would be released into the extracellular compartment. Flach *et al.* reported that most of the Alum is not internalized and no Aluminum was detected in the cell^23^. However, Hornung *et al.* observed phagocytosis of crystals and lysosomal acidification^21^. The exact mechanism by which Aluminum hydroxide engages GPRC6A remains to be identified but our results suggest a direct activation of the Nlrp3 inflammsome by Alum via GPRC6A.

In sharp contrast to the role of GPRC6A in innate immune responses to Alum, we observed elevated humoral responses to Alum when GPRC6A was absent. Both Th2 type immunity associated antibody isotypes IgG1 and IgE were found to be increased in response to Ova/Alum in GPRC6A−/− mice. The increased IgG1 production was even detectable in bone marrow cultures of GPRC6A−/− mice. This was not due to a general defect in humoral responses in GPRC6A−/− mice because immunization with an alternative adjuvant (CFA) resulted in normal Ova-specific IgG1 concentrations, and also the immunization with high dose Ova and no additional adjuvant showed comparable antibody concentrations in wildtype and GPRC6A−/− mice. The route of administration plays no role as both intraperitoneal and intramuscular injection of Alum/Ova resulted in increased Ova-specific IgG1 concentrations in GPRC6A−/− mice. The administration of Ova in combination with Aluminum lactate, which forms no gel, resulted in increased IgG1 production suggesting that free Aluminum ions are able to act as an adjuvant. Our studies were performed using Alum Imject®, which is a combination of Aluminum hydroxide and Magnesium hydroxide. Magnesium ions are also agonists of GPRC6A^17^, but to verify that the increased antibody concentrations in GPRC6A−/−mice are not due to the presence of Magnesium hydroxide, we used Aluminum hydroxide as single adjuvant and obtained the same results.

CaSR−/− mice were not available to us but we tested the involvement of one other Aluminum-sensing receptor CaSR using the specific inhibitor Calhex 231^18^. We also observed the increased IgG1 concentrations seen in GPRC6A−/− mice. However, the participation of CaSR in Alum-induced humoral responses remains to be analyzed in further studies.

In line with other studies^10^,^11^,^12^ we were also not able to connect the Alum-induced activation of the Nlrp3 inflammasome with humoral responses to Alum. Both Nlrp3−/− and ASC−/− mice showed normal IgG1 responses to Alum. Kool *et al.* reported a partial contribution of the Nlrp3 inflammasome showing reduced IgE concentration in Nlrp3−/− mice^9^. Interestingly, IgG1 concentrations were increased in Nlrp3−/− mice although not statistically significant^9^. We, however, observed normal IgE responses to Alum in Nlrp3−/− mice.

In GPRC6A−/− mice, IL-1β concentrations in the peritoneal cavity were diminished in response to Alum compared to wildtype mice. To assess if the missing IL-1β is responsible for the increased antibody production in GPRC6A−/− and to test the involvement of the inflammasome in humoral responses, we blocked IL-1 with IL-1Ra in Ova/Alum-immunized wildtype. The IgG1 responses were comparable between control and IL-1Ra-treated mice. This result, in combination with the results on Nlrp3−/− and ASC−/− mice, shows that the initial innate immune response to Alum resulting in IL-1 production, is not linked to the humoral response. IL-1 blockade was not studied in GPRC6A−/− mice because IL-1 is already diminished in response to Alum in these mice and the cytokine neutralization in wildtype mice should simulate this situation.

Since the increased antibody production seen in GPRC6A−/− mice is independent of the decreased inflammasome-dependent innate response in those mice, we speculated that GPRC6A might play a direct role in cells of adaptive immunity. We were able to show that Alum has a direct effect on IgG1 antibody production of B cells *in vitro*, as reported previously^20^. Importantly, we show for the first time that B cells do express GPRC6A, but no CaSR. B cells with missing GPRC6A produced higher antibody titers upon LPS/Alum stimulation *in vitro*, whereas proliferation and mobilization of activation marker were comparable to wildtype B cells. This increase in antibody production *in vitro* is in line with our *in vivo* results. However, Alum probably does not engage B cells directly *in vivo*, but it is possible that other GPRC6A ligands (aluminum ions, calcium ions, amino acids) are involved.

Aluminum-based adjuvants are known to elicit a Th2 type immune response associated with IgG1 and IgE antibody class switch^24^. CD4+ cells with a Th2 cytokine profile as well as TfH cells need to be activated to fulfill a prototypic antibody response with class switch. TfH frequencies were found unaltered in GPRC6A−/− mice. Ova-specific CD4+ T cell proliferation was similar in splenocytes from wildtype and GPRC6A−/− mice but typical Th2 cytokines like IL-4, IL-5 and IL-13 were found in increased concentrations in splenocyte cultures (restimulated with Ova) from GPRC6A−/− mice. This suggests that enhanced antibody titers from B cells are a result of enhanced Th2 immunity in GPRC6A−/− mice. One additional prototypic Th2 cytokine is IL-10. Beyond Th2, IL-10 is a pleiotropic cytokine with distinct functions in innate and adaptive immunity. IL-10 is produced by a number of different cell types, including innate myeloid cells as well as T cells and B cells. IL-10 produced by B cells at day 6 upon immunization of mice with Ova/Alum might promote a Th2 like phenotype in CD4+ T cells by suppressing Th1 polarization, as it has been proposed earlier^25^. IL-10-producing regulatory B cells (B10 cells) are able to downregulate inflammation and autoimmunity, and one proposed mechanism is the inhibition of Th1 differentiation^26^. Our findings of an increased IL-10 production of B cells at earlier timepoints and an increased Th2 phenotype with elevated IL-4, IL-5 and IL-13 in GPRC6A−/− splenocytes at later time points after immunization supports this idea. Maseda *et al.* showed that regulatory B cells are able to differentiate into plasma cells after transient IL-10 production^27^. One might speculate that GPRC6A plays a role in the differentiation/activation of regulatory B cells, which in turn induce a Th2 bias, but further data on the involvement of B10 cells are needed.

In our *in vitro* setting using isolated B cells, the LPS-induced release of IL-10 by B cells might be an anti-apoptotic signal and support plasma cell differentiation^28^. Whether B cells from GPRC6A−/− mice have survival advantages need to be analyzed in further studies. However, in addition to direct effects of GPRC6A on B cells involving IL-10, other mechanisms/cells might contribute to the enhanced antibody response when Alum is used as adjuvant, because blocking of CaSR also leads to increased antibodies concentrations, but murine B cells are deficient for this receptor. In line, Cheng *et al.* observed increased immune responses of dendritic cells and T cells in mice deficient of gut epithelial CaSR^29^, demonstrating that the local loss of CaSR on gut epithelial cells is able to influence systemic innate and adaptive immune responses.

In summary, our study proposes a dual role of GPRC6A in Alum adjuvanticity. It shows that Alum activates the Nlrp3 inflammasome via GPRC6A in the early innate immune response following immunization with the adjuvant. In contrast, the later humoral immune response is increased when GPRC6A is absent, which suggests that GPRC6A limits the humoral response to Alum.

## Methods

### Animals

GPRC6A−/− mice on C57BL/6 background were described previously^30^. ASC−/− and Nlrp3−/− mice^31^,^32^ were provided by Genentech (CA, USA). Caspase1−/− (NOD.129S2(B6)-Casp1^tm1Sesh^/LtJ) mice were from Jackson Laboratories. GPRC6A−/−, ASC−/− and Nlrp3−/− mice were bred in the animal facility at the Medizinisches Experimentelles Zentrum in Leipzig, Germany. C57/BL6 wildtype mice were either taken from littermates of the GPRC6A ko breeding or obtained from Charles River (Germany). All animal experiments were approved by and performed according to institutional guidelines of the animal ethics committee at the University of Leipzig.

### Antibodies, reagents

Alum (Imject^®^ Alum) from Pierce, Al-lactate from Sigma-Aldrich, Aluminum hydroxide from InvivoGen, CFA (Chondrex, USA), CFDA-SE (Molecular Probes, OR), IFA (Sigma-Aldrich) Ovaprotein (chicken, ovalbumin, grade V, Sigma-Aldrich), Calhex 231 was from Santa Cruz Biotechnology, Fc receptor block (aCD16/CD32) was from Miltenyi. Antibodies were obtained from Miltenyi Biotech (aCD3, aCD5, aCD11c, aCD49b, aCD19, aCD69, aCD86 aCD273 = PDL2, aMHC-II, aCD279 = PD1, aCXCR5, aSiglecF), ebioscience (aLy6C, aF480,aCD11b), R&D (aCCR2) Beckton Dickinson (aLy6G and aCD4) in different conjugates of FITC, Alexa488, PerCp, Pe and APC. Recombinant murine IL-4 was from Immunotools. Functional grade aCD40 for culture was obtained from ebioscience.

### *In vitro* cultures of peritoneal macrophages

Cells were isolated from the peritoneal cavity by lavage with 3 ml PBS. Cells were seeded in 1.5 × 10^5^/96 well and after 2 hours non-adherent cells were removed. Wells contained ~1 × 10^5^ macrophages per 96 well and were stimulated with LPS (100 ng/ml) and Alum (40 μg/ml) in RPMI supplemented with 10%FCS, 1% glutamin and 1% P/S.

### Immunization protocol

Mice were immunized at the age of 6–12 weeks into the peritoneal cavity (i.p.) using Ova (1 μg or 10 μg) combined with alum (1 mg), Aluminum hydroxide (1 mg) or Al-lactate (0.1 mg) or with high dose Ova (1 mg) without adjuvants. Immunization with Ova (1 μg) in CFA (0.2 mg) was performed s.c. on two injections sites. Intramuscular (i.m.) immunizations with Ova (1 μg) combined with alum (1 mg) were performed into both thighs. Ovaprotein was dissolved in PBS, sterile filtered and stored in -20°C until use. Ova-alum and Ova-CFA were both prepared according to the respective manufactures instructions. Ova-Al-lactate was prepared by slow addition of equal volume of Ova to Al-lactate dissolved in Na-acetat and sterile filtered before use. At day 10, mice were boosted i.p. s.c. or i.m. with half the dose of antigen, but equal amounts of aluminum adjuvant, CFA was replaced to IFA.

### Bone marrow cultures of immunized mice

Femur of both hind legs were prepared and flushed with PBS to obtain the bone marrow. Single cell suspension was obtained by passing the bone marrow through 70 μm filter. Cells were cultured in RPMI1640 with supplements at a density of 1 × 10^6^/well of 24 well plates. Supernatants were taken at day 5.

### Cellular peritoneal composition and TfH analyzes in splenocytes upon ova-alum injection

To identify recruited cells upon immunization with Ova/Alum from residential cells in the peritoneal cavity, the following staining protocol was applied. Mice were sacrificed 24 hrs post immunization by use of Isofluran anesthesia and subsequent CO_2_ inhalation. 3 ml of PBS were infused into the peritoneal cavity and recovered after 5–7 min. Recovered cells were counted and used for subsequent FACS analysis. Before the addition of the monoclonal antibodies, Fc-receptors were blocked by the use of aCD16/32. The following gating strategy was applied. First FSC/SSC gating included all visible cell populations. Second gate was implemented to exclude dead cells, according to propidium iodide negative cells. Third consecutive gate identified respective cell populations and provided the percentage of cells given in the graphs. For some cell populations, further subgatings were performed and are stated in detail when applied.

Global B cell population was defined by CD19 expression and was equal in frequencies in wildtype and GPRC6A−/− mice. Frequencies of B cells subpopulations refer to pregating on CD19+ cells and were identified with aCD5 and aCD11b (Mac1). This strategy enabled the dissection into conventional B cells (B2, CD5^−^CD11b^−^) and B1 B cells (CD11b^+^CD5^+^ or CD5^−^). Further subdivision into B1a (CD11b^+^CD5^+^) and B1b (CD5^−^CD11b^+^) was done from the same plot/gate. Tissue resident macrophages were identified by CD11b^+^F480^+^ expression. Eosinophiles (Eo’s) were identified by SiglecF^+^CD11b^+^F4/80^−^ expression and neutrophils (Neutro’s) by Ly6G^+^CD11b^+^ expression. DC’s were marked by CD11c^high^, NK cells by CD49b^+^CD3^−^ expression, T cells by CD3^+^ staining and NKT cells by CD3^+^CD49^+^ expression.

Splenic TfH cells were identified on day 21–25 post immunization with Ova/Alum by gating on live CD4+ lymphocytes followed by gating on PD1/CXCR5 double positive cells.

Before the addition of monoclonal antibodies, Fc-receptors were blocked by the use of aCD16/32. Dead cells were excluded by gating on propidium iodide negative cells.

Cells were measured by flow cytometry (Calibur and LSRII, both Beckton Dickinson) and analyzed with FlowJo.

### Cytokine detection from peritoneal cavity upon ova-alum immunization

Peritoneal lavage was prepared 4 and 24 hrs post immunization. For cytokine analyses, the cell free supernatant from the first centrifugation step was used and 1% of FCS was added prior to freezing. The volume of the supernatant was decreased 10 times by the use of Amicon Ultra-4 10K centrifugal devices (Millipore).

### B lymphocyte cultures from spleen and total splenocyte cultures of unimmunized and immunized mice

Spleens were isolated from mice. After preparation of a single cell suspension, cells were treated with erythrocyte lysis buffer. Total B cells were isolated by positive sorting using aCD19 beads or negative sorting with aCD43 beads, according to the Manufactures instructions (both Miltenyi). Cell proliferation was assayed by labeling pure B cells (92–98%) or total splenocyte cultures with CFDA-SE according to the manufactures guidelines. B cells and total splenocytes were plated into 96 well culture plates in RPMI1640 supplemented with 10% FCS, 1% P/S, 1% glutamin, 1% sodium pyruvate and 50 μM β-ME. B cells from unimmunized mice were stimulated with LPS (2.5 μg/ml) and IL-4 (10 ng/ml) with the addition of Alum (120 μg/ml) for the duration of 24 hrs (CD69 detection) or 7 days (Proliferation, CD86, Immunoglobulin detection). B cells from immunized mice (day 6 or 8) were cultured on Ova (200 μg/ml) precoated in 96 well plates in 5 × 10^5^/well and with the addition of soluble a-CD40 (10 μg/ml) for 5 days. Total splenocytes cultures with 5 × 10^5^/well in 96 well plates from immunized mice (day 21–25) were cultured with Ova (1 mg/ml) for 3 days.

### Cytokine specific ELISA’s and CBA (cytometric bead assay)

Cytokines from cell culture supernatants or peritoneal cavity were either analyzed by commercial available Elisa’s IL-1α (R&D Systems, sensitivity 2.5 pg/ml), IL-1β (cell culture supernatants OptEIA, BD Biosciences, limit of detection 15.6 pg/ml; peritoneal lavage Platinum ELISA, eBioscience, sensitivity 1.2 pg/ml), IL-10 (OptEIA, BD Biosciences, limit of detection 31.3 pg/ml), PGE_2_ (R&D Systems, sensitivity 41.4 pg/ml) or flow cytometry based CBA Kit’s (IL-4, -5, -6, -13, -21, IFNγ, MCP-1 and TNF) (limits of detection: IL-4 0.03 pg/ml, IL-5 5 pg/ml, IL-6 1.4 pg/ml, IL-13 2.4 pg/ml, IL-21 4.8 pg/ml, IFNγ 0.5 pg/ml, TNF 0.9 pg/ml, MCP-1 52.7 pg/ml) from Beckton Dikinson.

### *In vivo* blockade of IL-1R or CaSR in wildtype mice

IL-1R was blocked by the use of the IL-1 receptor antagonist Anakinra (Kineret®). Time points for Anakinra treatment were 30 min before immunization with Ova/alum, 24 hrs and 48 hrs thereafter (1 mg/mouse i.p. at each timepoint). The same procedure was applied at the time point of booster immunization on day 10. CaSR was blocked *in vivo* by the use of an allosteric inhibitor, Calhex 231. Calhex231 at 50 μM was applied 30 min before immunization with Ova/alum and 24 hrs thereafter. The solvent chloroform was used as control.

### Ova-specific and mouse immunoglobulin detection

Antibodies were analyzed from serum of unimmunized mice, immunized primary or secondary response or from B cell supernatants. Ova-specific antibodies of the IgM, IgG1, IgG2b, IgG3 and IgE isotype were analyzed with commercial available Kits from Biotrend (Cologne, Germany), following the manufactures instructions. Total mouse IgG1 from culture supernatants or serum was detected by self made ELISA. In brief, plates were coated with capture antibody (a-mouse IgG1 (SBA 1070-01, Southern Biotech) in 2.5 μg/ml over night. Plates were blocked with 0.1%gelatine/0.5%FBS for 2 hrs followed by sample incubation in different concentrations and standard mIgG1 (MOPC21, Beckton Dikinson) for 1.5 hrs. HRP labeled detection antibody anti mouse IgG1 (SBA 1070-05-HRP, Southern Biotech) was used 1:3000 and incubated for 2 hrs. Plates were developed with TMB and stopped with H_2_SO_4_, followed by measurement with 450 nm on a standard plate reader.

### Detection of CaSR and GPRC6A

Cell lysis, gel electrophoresis and western blot were performed as described previously^14^.

### Statistical analysis

For statistical analysis the software Sigma Stat was used. Normality test was performed and when data were normally distributed Student t-test was used and data were presented using bar charts and Mean  ±  SEM. When data were not normally distributed Mann Whitney test was applied and data were presented using boxes and whiskers (represent 25–75 and 5–95 percentiles).

## Additional Information

**How to cite this article**: Quandt, D. *et al.* GPRC6A mediates Alum-induced Nlrp3 inflammasome activation but limits Th2 type antibody responses. *Sci. Rep.* **5**, 16719; doi: 10.1038/srep16719 (2015).

## Supplementary Material

Supplementary Information
