# Supplementary material for: GPRC6A mediates Alum-induced Nlrp3 inflammasome activation but limits Th2 type antibody responses
Source: Sci Rep. 2015 Nov 25;5:16719. doi: 10.1038/srep16719 (PMC4658484; doi:10.1038/srep16719)
Supplement: Supplementary Information [file srep16719-s1.pdf]

GPRC6A mediates Alum-induced Nlrp3 inflammasome activation but limits Th2 type antibody responses

Dagmar Quandt, Kathrin Rothe, Christoph Baerwald, Manuela Rossol

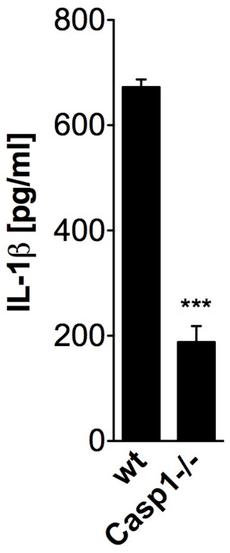

Supplementary Figure 1: Alum-induced IL-1β production depends on Caspase-1

Peritoneal macrophages from 3 wildtype (wt) and 3 Caspase-1-/- (Casp1-/-) mice were stimulated with Alum, and after 16h, the supernatant was tested for IL-1β using ELISA. Statistical analysis was performed using t-test. Bars represent mean±SEM. (*** $P<0.001$)

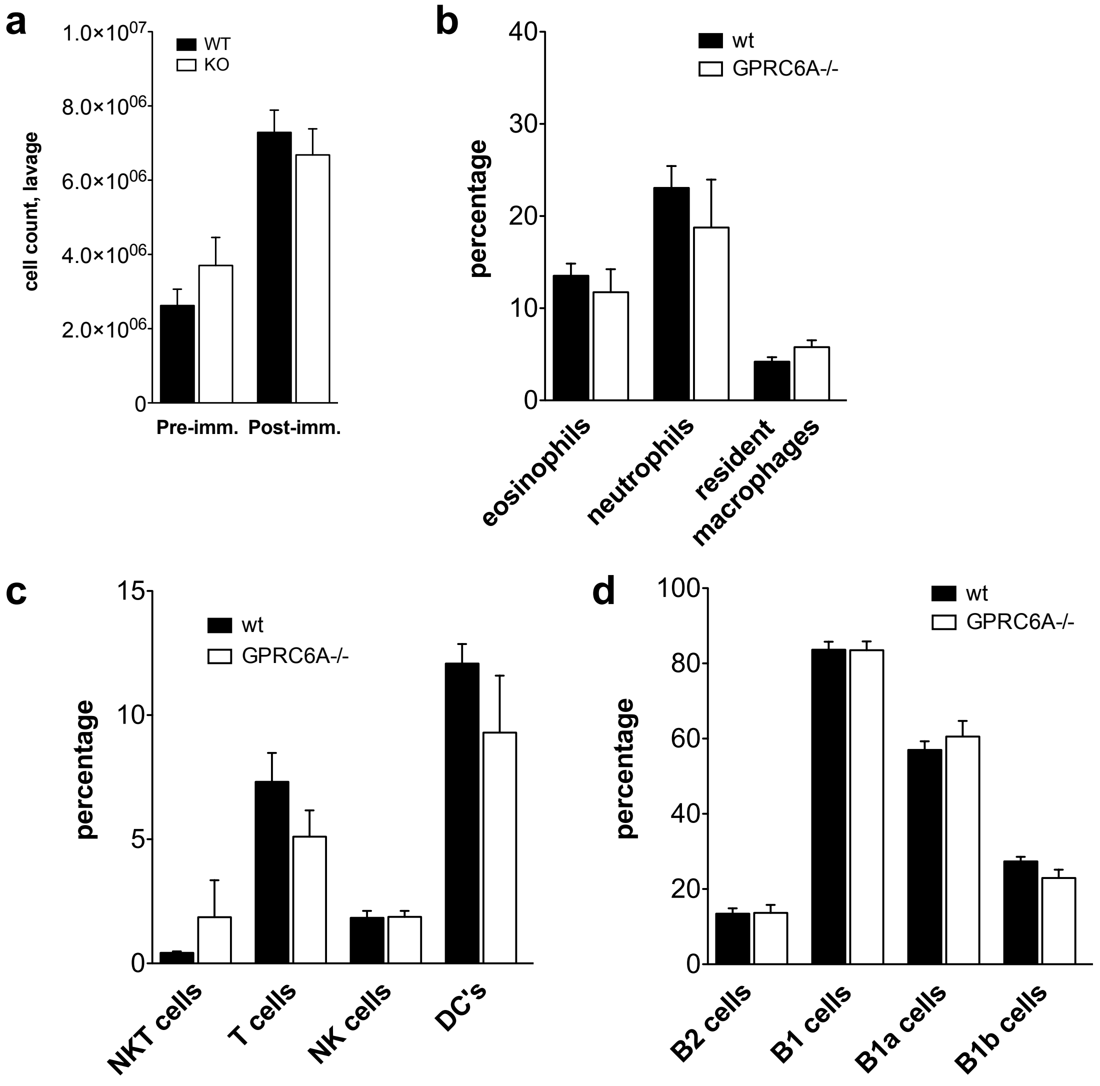

Supplementary Figure 2. Peritoneal cellular composition 24hrs after Ova/Alum i.p. immunization Respective cell populations were identified as described in M&M and analysed by flow cytometry. (a) Total cell count of peritoneal lavage of the peritoneal cavity, prior to and after Ova/Alum immunization. (b) Granulocytes (Eosinophils, Neutrophils) and tissue resident macrophages. (c) T cells, NK cell and NK T cells and DCs. (d) B cells (100%) pregated on CD19+ cells, subdivided into B2 and B1 with B1a and B1b. Data are from 2 independent experiments with a total of 6 wildtpye (wt) and 6 GPRC6A-/- mice. Statistical analysis was performed using t-test. Bars represent mean±SEM.

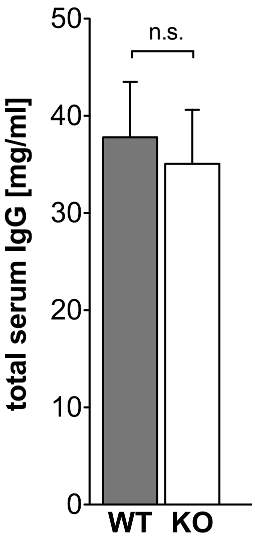

Supplementary Figure 3: Total serum IgG is not different in wildtype and GPRC6A-/- mice.

Total IgG was measured by ELISA in the serum from 10 wildtype (WT, gray) and 10 GPRC6A-/- (KO, white). Statistical analysis was performed using t-test. Bars represent mean±SEM.
